# Determination of Factors Influencing the Health Belief Model (HBM) and Adherence to Intravitreal Anti-vascular Endothelial Growth Factor (VEGF) Among Patients With Diabetic Macular Edema (DME)

**DOI:** 10.7759/cureus.34669

**Published:** 2023-02-06

**Authors:** Jacqueline Ting Yih Ling, Ayesha Mohd Zain, Ainal Adlin Naffi, Mushawiahti Mustapha, Wan Haslina Wan Abdul Halim

**Affiliations:** 1 Ophthalmology, Universiti Kebangsaan Malaysia Medical Centre, Kuala Lumpur, MYS; 2 Ophthalmology, KPJ (Kumpulan Perubatan Johor) Ampang Puteri Specialist Hospital, Kuala Lumpur, MYS

**Keywords:** dme, diabetic macular edema, hbm, diabetes, anti-vegf, health belief model, intravitreal

## Abstract

Background

Diabetic macular edema (DME) is becoming one of the leading causes of blindness worldwide with a significant impact on quality of life. The effectiveness of intravitreal (IVT) anti-vascular endothelial growth factor (VEGF) therapy has been established by clinical trials and has become the treatment of choice in the majority of DME patients in reducing macular edema and improving visual acuity. Frequent drop-out and discontinuation of treatment are major issues. Lack of compliance can lead to worsening outcomes and be a burden to patients and the healthcare system.

Purpose

The purpose of this study is to assess multiple factors that affect adherence to IVT anti-VEGF treatment among patients with DME, including socioeconomic causes and the Health Belief Model (HBM) domains, in addition to exploring the relationship between them.

Methods

This cross-sectional study was conducted among DME patients in Hospital Canselor Tuanku Muhriz, Kuala Lumpur, Malaysia, from December 2020 to June 2021. We identified eligible patients using a retrospective chart review of clinical findings and optical coherence tomography (OCT) findings. Included subjects were of Malaysian nationality, aged 18 years and above, who were initiated or re-initiated IVT anti-VEGF treatment regime and on follow-up for at least six months from initial injection from January 2019 onwards. A translated and validated self-administered questionnaire was given to the respondents. Data were analyzed using IBM SPSS Statistics for Windows, Version 26.0 (Released 2019; IBM Corp., Armonk, New York, United States). Demographics of the patient were summarized using descriptive statistics, independent sample t-test was used to compare the difference in components of the HBM questionnaire. Linear regression was further used to explore the relationship between patients’ demographics and the HBM component.

Results

A total of 141 patients participated in this study, of whom 56.2% patients were adherent to treatment. The majority were aged 60 years and above (56.7%), male (52.5%), Malay (38.9%), and married (71.6%). There was a significant statistical difference in patients who were adherent to treatment, in terms of life entourage (p=0.004, Fisher Exact test). HBM domains that influenced adherence to treatment included perceived severity, perceived barriers, perceived benefits, cues to action, and self-efficacy (p<0.05, independent sample t-test). Further, multiple logistic regression tests on sociodemographic factors and HBM domains after eliminating confounding factors narrowed down the significant variables to perceived susceptibility (p= 0.023), perceived benefits (p< 0.001), and self-efficacy (p< 0.001).

Conclusion

Patients’ adherence to IVT anti-VEGF is influenced by perceived susceptibility to complications from DME, perceived benefits to the treatment, and self-efficacy.

## Introduction

Diabetes mellitus (DM) is a major public health concern in Malaysia, proven by the increasing prevalence of DM as seen from the National Health and Morbidity (NHMS) survey [1]. The number of diabetic retinopathy (DR) and vision-threatening diabetic macular edema (DME) cases is expected to reach 191.0 million and 56.3 million, respectively, by 2030 worldwide [2]. DR was the cause of blindness in 10.4% of Malaysian elderly citizens, as seen in the National Eye Survey in Malaysia [3]. Furthermore, DME can occur at any stage of DR and is a common cause of vision loss [4]. A Malaysian study found that among diabetes patients who had follow-ups at ophthalmology clinics, 51.6% had DR while 26.7% had DME [4]. Globally, DME is also becoming one of the leading causes of blindness [5]. Increasing loss of visual acuity not only affects the quality of life but also affects work-related productivity and healthcare-related costs [5]. It is estimated that the total annual cost of diabetes in Malaysia is around 600 million USD [6].

The pathogenesis of DME is highly complex and not fully understood. DME is characterized by the accumulation of intraretinal fluid, primarily in the inner and outer plexiform layers and within the central portion of the retina [7]. It is caused by the breakdown of the blood-retinal barrier that results in the leakage of fluid and proteins into the macula, causing the macula to swell, which in turn affects visual function [7,8]. Meanwhile, ischemic drive can cause further upregulation and release of the vascular endothelial growth factor (VEGF) [9]. VEGF is a potent factor in increasing retinal vascular permeability besides promoting angiogenesis. VEGF is a critical player between angiogenesis and inflammation, therefore, reinforcing the use of anti-VEGF agents for the treatment of DME [9].

In the past decade, treatment for DME has evolved extensively. Focal and grid photocoagulation therapy used to be the only option but the introduction of intravitreal (IVT) anti-VEGF has proven to be effective and superior to laser treatment [10,11]. The efficacy and safety profile of anti-VEGF therapy has been established by several large clinical trials and has become the treatment of choice in the majority of DME patients [10,12,13]. Recent studies depicted significantly positive visual and anatomical results regarding the use of anti-VEGF for the treatment of DME [14-16]. Following the Malaysia Diabetic Macular Edema Consensus Guidelines 2021, initiation of anti-VEGF treatment requires a loading phase of three or more consecutive monthly injections [17], followed by a maintenance phase during which intervals between injections are titrated according to the patient's needs, based on clinical signs and optical coherence tomography (OCT) findings [13,18].

Despite knowing the benefits of the treatment and the importance of adherence to IVT anti-VEGF, frequent drop-out and discontinuation of treatment is a major issue globally [19]. Studies have proven that almost half the patients will drop out and a big proportion of patients do not receive the ideal monthly treatment either due to the accessibility of medication or monetary issues [20]. In addition, Habib et al. depicted that 21% of patients with DME dropped injections during the first year of treatment [21]. Abu-Yaghi et al. illustrated high compliance of 85% to injections during the first year of follow-up [22], while Angermann et al. reported a 51% adherence rate in the first two years [23]. Lack of compliance has proven to lead to a worse outcome and could be a burden not only to patients but also to the healthcare and economic sector [20].

The Health Belief Model (HBM) was developed in the 1950s and is one of the most widely used models to understand and explain health behaviors including adherence to treatment by patients [24]. The underlying concept of the HBM is that health behavior is determined by personal beliefs or perceptions about a disease and is outlined by six domains, namely: perceived susceptibility, perceived severity, perceived benefits, perceived barriers, cues to action, and self-efficacy [24-26]. Recently, it has been applied to other health issues including understanding intentions to coronavirus disease 2019 (COVID-19) vaccinations [27] and compliance with medical treatment [26]. The HBM framework was utilized by Habib et al. to develop a structured questionnaire to determine factors affecting adherence to treatment among DME patients [21].

To the best of our knowledge, current literature and studies on patients’ adherence to treatment assess sociodemographic factors or HBM domains individually. There might be a gap in assessing both areas that may be interrelated where sociodemographics might influence HBM domains as well. Data for Malaysia remain scarce due to a lack of local studies and a more comprehensive approach is needed. Identification and understanding of these factors can guide future treatment strategies and policy setting. The result from this study can also be used for healthcare budget planning in view of the high burden cost of diabetes mellitus.

Thus, this study aimed to assess all related factors that may affect adherence to IVT treatment among patients with DME. These include socioeconomic factors and HBM domains in addition to exploring the relationship between them.

## Materials and methods

Study population and methodology

This was a cross-sectional study, conducted at the ophthalmology clinic in Hospital Canselor Tuanku Muhriz (HCTM), Kuala Lumpur, Malaysia, from December 2020 to June 2021. All the patients fulfilling the inclusion and exclusion criteria were invited to join the study and recruited via convenient sampling.

The targeted subjects were DME patients of Malaysian nationality, 18 years old and above, who were initiated or re-initiated IVT anti-VEGF treatment regime and on follow-up for at least six months from the initial injection, from January 2019 onwards. Exclusion criteria included patients who were unable to understand English or Malay language, with medical conditions that may impair their ability to communicate or respond logically, with incomplete or untraceable medical records, and whose follow-up and appointment were postponed during the COVID-19 Movement Control Order.

All procedures in this study adhered to the Declaration of Helsinki and Malaysian Guidelines for Good Clinical Practice (GCP). The study was approved by the Universiti Kebangsaan Malaysia (UKM) Research and Ethics Committee (Approval number: JEP-2020-683). Patients were fully informed and written informed consent was obtained. They were counseled on the research topic and its benefits and risks involved in the research and confidentiality of data. They were informed that their participation is voluntary, that they can withdraw anytime, and that there is no compensation from this research.

Questionnaire

The questionnaire used was from the study by Habib et al. to identify factors affecting compliance with follow-up and IVT anti-VEGF injection [18]. Permission was obtained from the author for translation and use in this study. The questionnaire has 26 items and was constructed to assess patients’ perception towards anti-VEGF treatment in DME based on six domains; perceived susceptibility (three items), perceived severity (three items), perceived benefits (four items), perceived barriers (nine items), cues to action (five items) and self-efficacy (two items). For perceived severity, susceptibility, benefits, and barriers, responses were recorded on a five-point Likert scale ranging from 1 (strongly disagree) to 5 (strongly agree). For cues to action, responses were in the form of dichotomous questions, Yes and No, with a score of 1 for Yes and 0 for No. For self-efficacy, a five-point Likert scale was used ranging from 1 (strongly impossible) to 5 (very possible). The total sum of scores for each domain was calculated for mean and standard deviation.

The original validated English questionnaire was translated into Malay using international guidelines for cross-cultural adaptation to ensure the quality of the translated version and its consistency of meaning to the original version [28]. The content validity index (CVI) and face validity index (FVI) was 0.91 and 0.89, respectively, for all the domains after improvements were made based on the experts’ suggestions. Reliability testing was done on 30 respondents. Cronbach’s alpha value range between 0.84-0.93 was obtained for each of the domains and considered adequate for validation of the questionnaire to be used in this study [28].

A total of 180 eligible patients were identified but only 141 responded and participated in this study. Patients were identified using a retrospective chart review of all patients diagnosed with DME based on clinical and OCT findings. Information was obtained from the treatment logbook. The investigator was masked about the adherence of the patient to the IVT anti-VEGF treatment. In a clinical setting, each respondent was given a validated self-administered questionnaire. Respondents could choose to answer the English or Bahasa Melayu version of the questionnaire. They were given 25 minutes to answer the questionnaire.

Demographic data including age, gender, ethnicity, marital status, education level, occupation, life entourage, and financial source for IVT anti-VEGF were recorded. Information on adherence to treatment and follow-up was obtained from the medical record and treatment log sheet. Patients were subsequently divided into two categories, namely adherent and non-adherent. Patients were considered adherent if compliant to IVT anti-VEGF six monthly loading doses and maintained follow-up for a minimum of six months including injections during the period if indicated. Meanwhile, patients who failed the above condition are considered non-adherent.

Statistical analysis

Data were cleaned and analyzed using IBM SPSS Statistics for Windows, Version 26.0 (Released 2019; IBM Corp., Armonk, New York, United States). The distribution of numerical data was assessed using skewness, kurtosis, and histogram. Continuous variables were presented using mean and standard deviation if the data were normally distributed, otherwise median and interquartile range (25th percentile, 75th percentile). Categorical variables were presented as frequency and percentage.

Demographics of the patients were summarized using descriptive statistics, and differences in demographics between adherent vs non-adherent patients were compared using the Mann-Whitney U test, Pearson chi-squared test, and Fisher exact test. An Independent sample t-test was used to compare the difference in components of the HBM questionnaire between adherent vs non-adherent patients as well.

The components of the HBM questionnaire associated with adherence were further tested using logistic regression. Simple logistic regression was used as a univariable analysis to explore the relationships, and variables with a p-value less than 0.200 in univariable analysis were included in the variable selection process in a multivariable model. Variable selection forward logistic regression method was used. Multicollinearity and interaction terms were checked for the final model, while the model fit was assessed using the Hosmer Lemenshow goodness of fit test, classification table, and area under the receiver operating characteristic (ROC) curve.

The relationship between patients’ demographics and with HBM component was explored using linear regression. Simple linear regression was used as a univariable analysis to first explore the relationships, and variables with a p-value less than 0.200 in the univariables analysis were included in the variable selection process with a stepwise method. Multicollinearity, interaction, and heterocedasticity of the multivariable model were checked.

## Results

A total of 141 patients participated in this study. Table 1 depicted the sociodemographic characteristics of the patients. The majority of them were aged 60 years and above (56.7%), male (52.5%), Malay (38.9%), and married (71.6%); 26.9% of the patients live with their spouse while another 26.9% live with their children. With regard to education, 51.7% of them had reached the secondary level. The majority of them used their personal savings (45.5%) as the financial source; 30.5% were pensioners followed by 20.6% who are unemployed

**Table 1:** Sociodemographic characteristics of patients

Sociodemographic characteristics	n (%)
Age (years)	
≤ 30	1 (0.7)
31- 40	4 (2.8)
41 -50	18 (12.8)
51-60	38 (27.0)
>61	80 (56.7)
Gender	
Female	67 (47.5)
Male	74 (52.5)
Ethnicity	
Malay	69 (38.9)
Chinese	43 (30.5)
Indian	29 (20.6)
Others	0
Marital Status	
Single	21 (14.9)
Married	101 (71.6)
Divorced	2 (1.4)
Widowed	17 (12.1)
Education level	
No formal education	6 (4.3)
Primary	30 (21.3)
Secondary	73 (51.7)
Tertiary	32 (22.7)
Occupation	
Pensioner	43 (30.5)
Self-employed	21 (14.9)
Government	22 (15.6)
Private	26 (18.4)
Unemployed	29 (20.6)
Others	0
Life entourage	
With spouse	38 (26.9)
With children	38 (26.9)
Spouse and children	44 (31.2)
Alone	18 (12.8)
Others	3 (2.2)
Financial Source	
Personal	64 (45.4)
Government	50 (35.5)
Insurance	7 (5)
Others	20 (14.1)

Our study showed that 56.2% of the patients were adherent to treatment as opposed to 43.8% who were non-adherent and there was a significant association between life entourage and adherence (p= 0.004) (Table 2).

**Table 2:** Sociodemographics and adherence to treatment ^a^Pearson chi-square test; ^b^Fisher Exact test

Sociodemographic characteristics	Adherence		
			Adherent	Non-adherent	p-value
	n= 68 (56.2%)	n = 53 (43.8%)	
	Age (years)			
≤ 30	0	1	
31- 40	2	2	0.888^b^
41 -50	11	7	
51-60	20	18	
>61	46	34	
Gender			
Female	45	29	0.240^a^
Male	34	33	
Ethnicity			
Malay	43	26	
Chinese	22	21	0.328^a^
Indian	14	15	
Others	0	0	
Marital Status			
Single	9	12	
Married	60	41	0.270^b^
Divorced	2	0	
Widowed	8	9	
Education level			
No formal education	1	5	
Primary	13	16	0.125^b^
Secondary	45	28	
Tertiary	20	13	
Occupation			
Pensioner	24	19	
Self-employed	13	9	
Government	13	9	0.891^a^
Private	16	10	
Unemployed	13	15	
Others	0	0	
Life entourage			
With spouse	17	21	
With children	25	13	0.004^b^
Spouse and children	30	15	
Alone	4	13	
Others	3	0	
Financial Source			
Personal	31	33	
Government	29	21	0.113^b^
Insurance	4	3	
Others	15	5	

Table 3 demonstrates the relationship between various HBM domains and treatment adherence, Perceived barriers were significantly lower in adherent patients compared to non-adherent patients. Adherent patients were observed to have higher perceived severity, perceived susceptibility, perceived benefits, cues to action, and self-efficacy.

**Table 3:** Association between HBM domains and treatment adherence HBM: health belief model *Independent Sample t test

HBM domain	Adherent (mean ± SD)	Non-adherent (mean ± SD)	p-value*
Perceived severity	11.51 ± 1.75	10.52 ± 2.62	0.012
Perceived susceptibility	11.57 ± 2.34	10.74 ± 2.83	0.059
Perceived benefits	17.70 ± 2.02	13.34 ± 2.57	<0.001
Perceived barriers	15.33 ± 3.65	20.66 ± 5.76	<0.001
Cues to action	1.61 ± 1.21	0.90 ± 0.76	<0.001
Self-efficacy	8.94 ± 1.29	6.42 ± 1.24	<0.001

Simple and multiple logistic regression was subsequently conducted and summed up in Table 4. The variables which were significant in the univariable analysis included perceived severity (p= 0.010), perceived benefits (p< 0.001), perceived barriers (p< 0.001), cues to action (p< 0.001), and self-efficacy (p< 0.001), and were similar to Table 3 results. Nonetheless, the variables which were significant in the final model after eliminating the confounding effects included perceived susceptibility (p= 0.023), perceived benefits (p< 0.001), and self-efficacy (p< 0.001).

**Table 4:** Logistic regression of the HBM domains Multicollinearity and interaction were checked and not found. Hosmer Lemenshow goodness of fit test (p= 0.300); classification table (overall correctly classified percentage: 88.7%); area under ROC curve: 95.7% HBM: health belief model

HBM domain	Simple logistic regression			Multiple logistic regression		
	OR	95% CI	p-value	Adjusted OR	95% CI	p-value
Perceived severity	1.24	1.05, 1.46	0.01			
Perceived susceptibility	1.14	0.99, 1.30	0.062	0.75	0.58, 0.96	0.023
Perceived benefits	2.19	1.70, 2.81	<0.001	1.91	1.41, 2.59	<0.001
Perceived barriers	0.78	0.71, 0.86	<0.001			
Cues to action	1.99	1.37, 2.90	<0.001			
Self- efficacy	4.06	2.58, 6.39	<0.001	3.03	1.85, 4.98	<0.001

We used linear regression test to explore the relationship between each HBM domain and sociodemographic factors. Table 5 summarizes and demonstrates that education, life entourage, and financial source have effects on perceived benefits, life entourage on perceived barriers, and cues to action, respectively. Meanwhile, occupation, life entourage, and financial source affect self-efficacy.

**Table 5:** Relationship of HBM and signficant demograghic factor HBM: health belief model

HBM domain	Demographics
Perceived severity	
Perceived susceptibility	
Perceived benefits	Education
	Life entourage
	Financial source
Perceived barriers	Life entourage
Cues to action	Life entourage
Self- efficacy	Occupation
	Life entourage
	Financial source

Patients who completed secondary school (coef (95%CI): 1.78 (0.61, 2.95); p= 0.003) and tertiary education (coef (95%CI): 1.95 (0.48, 3.42); p= 0.010) had higher perceived benefits compared to patients with no formal education. Apart from that, patients who live with children [coef (95% CI): 1.24 (0.02, 2.46); p= 0.047], spouse and children (coef (95%CI): 1.50 (0.29, 2.71); p= 0.015) and others (coef (95%CI): 4.55 (1.17, 7.92); p= 0.009) were observed to have higher perceived benefits than those who live alone. Patients whose financial source came from the government (coef (95%CI): 1.23 (0.15, 2.32); p= 0.026) and others (coef (95%CI): 1.90 (0.38, 3.42); p= 0.015) has higher perceived benefits as well (Table 6).

**Table 6:** Relationship between education, life entourage, and financial source with perceived benefits

	Simple linear regression			Multiple linear regression		
	Coef	95% CI	P-value	Coef	95% CI	P-value
Education						
No formal education	Ref			Ref		
Primary	1.5	-1.20, 4.20	0.274	-	-	-
Secondary	3.05	0.49, 5.62	0.02	1.78	0.61, 2.95	0.003
Tertiary	3.15	0.46, 5.83	0.022	1.95	0.48, 3.42	0.01
Life entourage						
With spouse	Ref			Ref		
With children	1.65	0.23, 0.98	0.022	1.24	0.02, 2.46	0.047
Spouse &children	1.8	0.48, 3.13	0.008	1.5	0.29, 2.71	0.015
Alone	-0.04	-1.79, 1.71	0.967	-	-	-
Others	4.32	0.72, 7.91	0.019	4.55	1.17, 7.92	0.009
Financial source						
Personal	Ref			Ref		
Government	1.41	0.26, 2.56	0.017	1.23	0.15, 2.32	0.026
Insurance	0.11	-2.31, 2.54	0.928	-	-	-
Others	1.72	0.16, 3.28	0.031	1.9	0.38, 3.42	0.015

Patients who lived alone were found to have higher perceived barriers compared to patients who live with spouses (coef (95%CI): 4.45 (1.79, 7.11); p= 0.001) (Table 7).

**Table 7:** Relationship between life entourage with perceived barriers

	Simple linear regression			Multiple linear regression		
	coef	95% CI	P-value	coef	95% CI	P-value
Life entourage						
With spouse	Ref			Ref		
With children	-1.34	-3.70, 1.02	0.263	-	-	-
Spouse &children	-1.42	-3.69, 0.84	0.216	-	-	-
Alone	3.43	0.43, 6.43	0.025	4.45	1.79, 7.11	0.001
Others	-3.82	-9.99, 2.34	0.222	-	-	-

Patients who live with others such as in nursing homes or with relatives had higher cues to action compared to those who live with spouses only (coef (95% CI): 1.40 (0.15, 2.64); p= 0.028) (Table 8).

**Table 8:** Relationship between life entourage with cues to action

	Simple linear regression			Multiple linear regression		
	coef	95% CI	P value	coef	95% CI	P value
Life entourage						
With spouse	Ref			Ref		
With children	0.42	-0.07, 0.91	0.091	-	-	-
Spouse &children	0.21	-0.26, 0.68	0.378	-	-	-
Alone	0.04	-0.58, 0.66	0.902	-	-	-
Others	1.59	0.31, 2.87	0.015	1.4	0.15, 2.64	0.028

We used logistic regression to explore the factors associated with adherence. The variables significant in the univariable analysis simple logistic regression include perceived severity (p= 0.010), perceived benefits (p< 0.001), barriers (p< 0.001), cue to action (p< 0.001), and self-efficacy (p< 0.001). The variables with p< 0.200 were included in the variable selection process, and the variables significant in the final model were perceived susceptivity (p= 0.023), perceived benefits (p< 0.001), and self-efficacy (p< 0.001).

It was found that a unit increase in perceived susceptibility will decrease the odds for adherence by 25% (adjusted OR (aOR) (95% CI): 0.75 (0.58, 0.96); p= 0.023). On the other hand, a unit increase in perceived benefits was observed to increase the odds for adherence by 91% (aOR (95% CI): 1.91 (1.41, 2.59); p< 0.001). Moreover, it was observed that a unit increase in the self-efficacy score will increase the odds for adherence by 3.03 times (aOR (95% CI): 3.03 (1.85, 4.98); p< 0.001). The Nagelkerke's R squared of the final model was 0.759, indicating that 75.9% of the variation in adherence was explained by the three factors in the final model (Table 9). 

**Table 9:** Logistic regression for socio-economic factors and HBM domains against adherence Negelkerke's R squared = 0.759 Hosmer Lemenshow goodness of fit test (p= 0.300); classification table (overall correctly classified percentage: 88.7%); area under ROC curve: 95.7% HBM: health belief model; ROC: receiver operating characteristic

		Simple Logistic Regression				Multiple Logistic Regression		
	coef	OR	95% CI	P-value	Adjusted coef	Adjusted OR	95% CI	P-value
Age	0.01	1.01	0.98, 1.04	0.585				
Gender								
Male	Ref							
Female	-0.41	0.66	0.34, 1.30	0.23				
Ethnicity								
Malay	Ref							
Chinese	-0.46	0.63	0.29, 1.37	0.246				
Indian	-0.57	0.56	0.24, 1.36	0.201				
Marital status								
Single	Ref							
Married	0.67	1.95	0.75, 5.05	0.168				
Divorced	21.49	2.15x10^9^	0.00, ∞	>0.950				
Widowed	0.17	1.19	0.33, 4.29	0.796				
Education								
No formal education	Ref							
Primary	1.47	4.38	0.46, 42.08	0.201				
Secondary	2.08	8.04	0.89, 72.40	0.063				
Tertiary	1.99	7.31	0.76, 70.03	0.085				
Occupation								
Pensioner	Ref							
Self-employed	0.05	1.06	0.37, 3.03	0.92				
Government	0.13	1.14	0.40, 3.24	0.801				
Private	0.24	1.27	0.47, 3.42	0.641				
Unemployed	-0.3	0.74	0.29, 1.90	0.53				
Life entourage								
With spouse	Ref							
With children	0.87	2.38	0.94, 6.00	0.067				
Spouse and children	0.9	2.47	1.01, 6.02	0.047				
Alone	-0.97	0.38	0.11, 1.38	0.142				
Others	21.41	2.00x10^9^	0.00, ∞	>0.950				
Income source								
Personal	Ref	Ref		0.230				
Government	0.39	1.47	0.70, 3.10	0.311				
Insurance	0.35	1.42	0.29, 6.86	0.663				
Others	1.16	3.19	1.04, 9.83	0.043				
Perceived severity	0.21	1.24	1.05, 1.46	0.01								
Perceived susceptibility	0.13	1.14	0.99, 1.30	0.062	-0.29	0.75	0.58, 0.96	0.023
Perceived benefits	0.78	2.19	1.70, 2.81	<0.001	0.65	1.91	1.41, 2.59	<0.001
Perceived barriers	-0.25	0.78	0.71, 0.86	<0.001				
Cues to action	0.69	1.99	1.37, 2.90	<0.001				
Self-efficacy	1.4	4.06	2.58, 6.39	<0.001	1.11	3.03	1.85, 4.98	<0.001

## Discussion

To the best of our knowledge, this study was the first to explore the relationship between sociodemographic factors and HBM domains to adherence to IVT anti-VEGF among those with DME. In 2021, Wong et al. reported a non-compliance rate of 35.1% in a small study cohort and assessed the socio-economic factors affecting non-adherence to treatment in a private center in Melaka [29]. Meanwhile, our study depicted a higher non-adherence rate of 43.8%.

Adherent patients were observed to have higher perceived severity, perceived susceptibility, perceived benefits, cues to action, and self-efficacy. These observations are quite similar to the findings by Habib et al. [21].

Patients with higher perceived susceptibility are more concerned about the loss of vision secondary to DME. When combined with a higher perception of disease severity, they have a stronger effect on the intention to treatment [27]. Zampetakis et al. also demonstrated this synergistic effect in their study using HBM to predict vaccination intention [27]. This highlights the importance to increase the risk perception and severity among the patients, especially among those who perceive the disease as being non-sight-threatening.

Perceived benefits were also affected by education level, life entourage, and financial sources. Both Abu-Yaghi et al. [22] and Habib et al. [21] showed contrasting results that lack of education was not found to be significantly affecting compliance with treatment. In Malaysia. Wong et al. reported that there is considerable evidence to suggest that education level is an important social determinant to support and access health services and information [30]. We postulated that the possible reason is that most health education including pamphlets and social media videos regarding IVT anti-VEGF are mostly in English. Thus, patients who do not receive formal education and cannot understand English have less access to treatment information.

Regarding cues to action and self-efficacy, the findings of the current study are quite similar to the ones reported by Reiter et al. [31] suggesting providers’ recommendation is key in promoting health behavior. Otherwise, the most prevalent cues that especially drove patients’ compliance were when friends and relatives shared their positive experiences [32]. Another important point to highlight is the effect of financial source on self-efficacy. Wong et al. demonstrated that financial constraint was the commonest reason for non-compliance to IVT anti-VEGF [29]. Meanwhile, Muller et al. also identified financial cost as a challenge for DME patients in Germany [33]. In our study, treatment cost has clearly been shown to influence patients’ confidence in maintaining regular IVT treatment and follow-up. Our patients were mainly self-paying or subsidized groups under Jabatan Perkhidmatan Awam (JPA). Where there are out-of-pocket expenses, high drug cost leads to poor treatment adherence among our patients [34].

Furthermore, other domains also play a minimal influential role in affecting adherence to treatment. When perceived barriers to getting IVT anti-VEGF were low, it had a positive effect on adherence to treatment. This finding is comparatively similar to the study by Habib et al. [21]. Examples of barriers assessed in this study include the experience of injection, disbelief in injection, time, travel and financial cost, difficulties in remembering appointments, and also the psychological burden of the accompanying person. Wong et al. depicted that financial constraints and logistic difficulties contributed 38% and 19%, respectively, to the dropout rate from the IVT treatment regime in Melaka [29]. Other studies have also identified similar barriers to treatment adherence, including time and financial burden and disbelief in treatment [35]. Otherwise, the discomfort-associated experience of pain and fear are also commonly reported, similar to findings by Habib et al. [21].

The World Health Organization (WHO) has outlined non-adherence as a major issue in the care of chronic illness [36]. Thus, it is pertinent to identify the contributing factors and outline suggestions to improve the adherence rate. From the results of this study, we would like to suggest ways to improve adherence via increasing perceived susceptibility, perceived benefits, and self-efficacy.

Effective counseling towards patients and their families to improve understanding of the disease itself, the benefits of treatment, and the consequences of non-adherence to treatment. Counseling should be done not only in the ophthalmology clinics but also can be reinforced in a multidisciplinary manner by including other healthcare counterparts involved in the care of diabetic patients. Recommendations and assurance provided by doctors and peers do influence patients’ behavior [34].

Information should ideally be available in English, Malay, Chinese, and Tamil in easy-to-understand language. It should be widely available on various media platforms for easy access. Otherwise, common barriers include discomfort during injection and anxiety, which can be addressed via assurance before and during the procedure. Moreover, financial cost has always been an issue for those of lower income. Hence, policies can be looked into to lower the cost of IVT anti-VEGF.

In addition, we also see the opportunity for the use of this validated questionnaire in future studies in other eye diseases including adherence to IVT anti-VEGF in age-related macular degeneration.

Nevertheless, there are a few limitations to this study. First, the developer of the original questionnaire was not involved in the translation and validation process. The translated version of the questionnaire may not be a perfect reflection of the original English version and could introduce bias in the results. Second, this is a single-center study and the result may not be representative of the whole nation. Otherwise, the convenience sampling method used in this study may not provide a representative result. Third, when answering the questionnaire, some patients were assisted by family members in recalling information, which can result in recall bias. In addition, the self-administered format of the questionnaire may introduce bias as participants may have different levels of comfort with answering questions and may have varying literacy levels. The 25-minute time given may be too short for some participants to reflect on their responses, leading to potentially biased answers.

## Conclusions

There are multiple factors that influence adherence to IVT anti-VEGF treatment among DME patients. These factors include sociodemographic factors and HBM. However, the most significant factors are HBM domains; perceived susceptibility to complications from DME, perceived benefits to IVT anti-VEGF treatment, and self-efficacy, which is confidence to adhere to treatment. Identifying these factors allow better understanding and outline suggestions to improve adherence. Life entourage is also an important factor that affects the significant HBM domains. This study can be a guide to future strategies to improve treatment adherence. It will also be useful in setting new treatment policies, contributing to better treatment outcomes. 
